# Supplementary material for: GmFAD3A, A ω-3 Fatty Acid Desaturase Gene, Enhances Cold Tolerance and Seed Germination Rate under Low Temperature in Rice
Source: Int J Mol Sci. 2019 Aug 3;20(15):3796. doi: 10.3390/ijms20153796 (PMC6696117; doi:10.3390/ijms20153796)
Supplement: Supplementary file 1 [file ijms-20-03796-s001.pdf]

International Journal of
Molecular Sciences

Supplementary Materials:

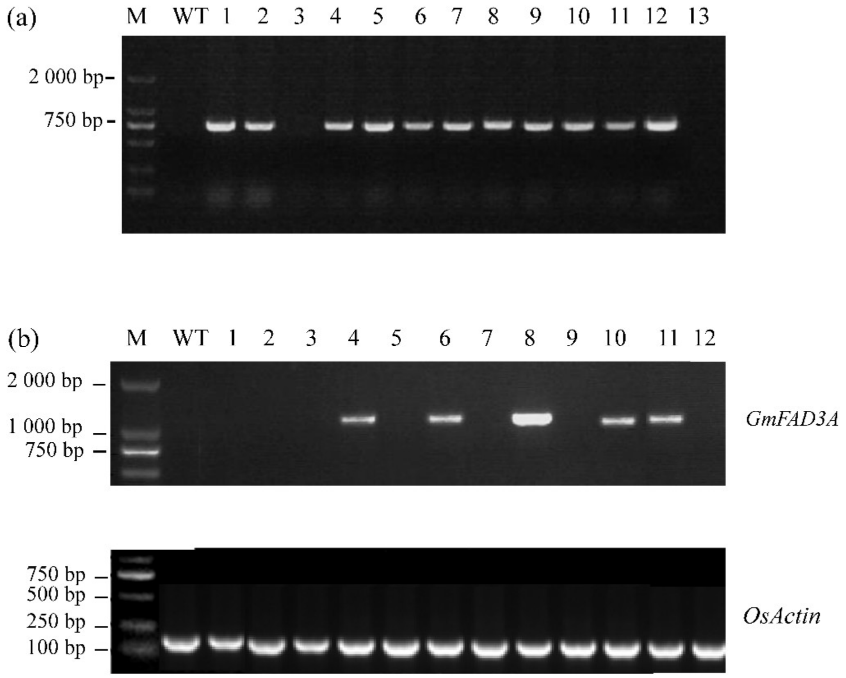

Figure S1. Analysis of T_0 transgenic rice. a, *Hpt* PCR detection of T_0 transgenic rice. b, The *GmFAD3A* expression in transgenic plants was determined by RT-PCR analysis.

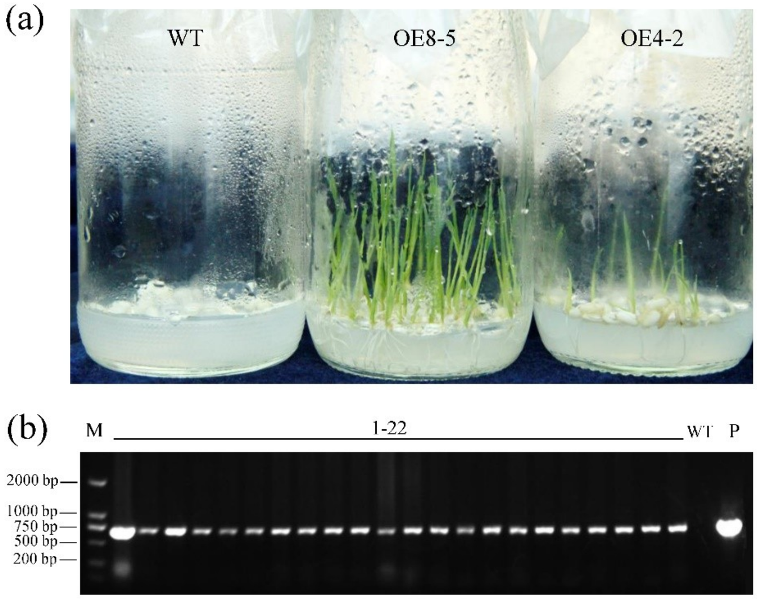

Figure S2. Screening of homozygous plants. A, The hygromycin-resistant plants were selected to further plant. B, *Hpt* PCR detection of OE8-5 transgenic rice.

Table S1. Primers for *GmFAD3A* functional analysis.

Purpose	Name	Sequence (5'→3')
GmFAD3A cDNA clone	FAD3-cF	CGCGGATCCATGGTTAAAGACACAAAGCC
	FAD3-cR	CGCGGATCCGAAACTCAGTCTCGGTGCGAG
Transgenic analysis	HptF	CTGAACTCACCGCGACGTCTGTC
	HptR	TAGCGCGTCTGCTGCTCCATACA
qRT-PCR analysis	OsACT1-qF	TCTCTCTGTATGCCAGTGGTCGT
	OsACT1-qR	TCATAGTCCAGGGCGATGTAGG
	GmFAD3-qF	GCAATCTGTTCCCACCCAGT
	GmFAD3-qR	TCCATTCCTTGCCGCGATAC

Table S2. Analysis of yield parameters of WT and *GmFAD3A* OE lines.

Plants	WT	OE4-2	OE8-5
Total spikelets	117.1±1.42	109.3±2.33	116.3±2.36
1000-grain weight (g)	25.40±1.16	24.20±1.01	23.63±0.67*
Seed setting (%)	89.38±2.11	86.99±4.21	87.54±4.85
Grain length (mm)	7.88±0.21	7.52±0.13	7.72±0.34
Grain width (mm)	3.40±0.06	3.43±0.12	3.25±0.09
